# Synaptic Clustering and Memory Formation

**DOI:** 10.3389/fnmol.2019.00300

**Published:** 2019-12-06

**Authors:** George Kastellakis, Panayiota Poirazi

**Affiliations:** Institute of Molecular Biology and Biotechnology (IMBB), Foundation for Research and Technology Hellas (FORTH), Heraklion, Greece

**Keywords:** synaptic clustering, engram, dendrites, in-branch localization, memory

## Abstract

In the study of memory engrams, synaptic memory allocation is a newly emerged theme that focuses on how specific synapses are engaged in the storage of a given memory. Cumulating evidence from imaging and molecular experiments indicates that the recruitment of synapses that participate in the encoding and expression of memory is neither random nor uniform. A hallmark observation is the emergence of groups of synapses that share similar response properties and/or similar input properties and are located within a stretch of a dendritic branch. This grouping of synapses has been termed “synapse clustering” and has been shown to emerge in many different memory-related paradigms, as well as in *in vitro* studies. The clustering of synapses may emerge from synapses receiving similar input, or *via* many processes which allow for cross-talk between nearby synapses within a dendritic branch, leading to cooperative plasticity. Clustered synapses can act in concert to maximally exploit the nonlinear integration potential of the dendritic branches in which they reside. Their main contribution is to facilitate the induction of dendritic spikes and dendritic plateau potentials, which provide advanced computational and memory-related capabilities to dendrites and single neurons. This review focuses on recent evidence which investigates the role of synapse clustering in dendritic integration, sensory perception, learning, and memory as well as brain dysfunction. We also discuss recent theoretical work which explores the computational advantages provided by synapse clustering, leading to novel and revised theories of memory. As an eminent phenomenon during memory allocation, synapse clustering both shapes memory engrams and is also shaped by the parallel plasticity mechanisms upon which it relies.

## Introduction

### Memory Engram Allocation in the Brain

Memory formation and storage relies on structural changes that occur in the connectivity between neurons. Richard Semon, an early advocate of a physical theory of memory, was the first to term the neural substrate containing memories as the *memory engram* (Semon, 1904; Schacter, 2001). He defined the *engram* as the lasting modification produced by experience and stimulation in the brain. Attempts in the 20th century to find and identify the engram were intense. Karl Lashley is most famous for performing a long series of lesion experiments in the cerebral cortex of rats in an attempt to find associations between carefully targeted lesions and the ability of animals to solve maze tasks in which they were trained. While the lesions did cause memory impairments, Lashley’s studies showed that impairments occurred irrespective of the location of the lesion, leading him to conclude that the memory engram is not localized, but it is spread broadly and indiscriminately throughout the brain (Lashley, 1950). Other experiments identified specific loci of stimulation that evoke memories, such as the experiments of Penfield and Rasmussen who applied electrical stimulation to epileptic patients in order to identify the centers of seizures. They found that electrical stimulation in specific brain regions could cause a vivid recall of various memories (Penfield and Rasmussen, 1950). Another historically significant case is the famous patient Henry Molaison whose bilateral removal of the medial temporal cortex led to anterograde amnesia. This bolstered the idea that episodic memories may be processed in the hippocampus (Scoville and Milner, 1957) before being consolidated. These earlier studies hinted at the existence of cellular memory engrams but lacked the tools to visualize or manipulate them. Thus, a causal link between memories and the neural substrate that contains them remained elusive for decades.

It was not until a few years ago that optogenetics, molecular labeling methods and 2-photon imaging allowed scientists to precisely identify the neurons which are involved in the learning and recall of specific memories (reviewed in Rogerson et al., 2014; Josselyn et al., 2015; Tonegawa et al., 2015; Poo et al., 2016). The first study that revealed the cellular memory engram, i.e., the existence and identification of populations of neurons that are sufficient and necessary for learning was (Han et al., 2007). Since then, numerous studies have characterized the properties and even manipulated cellular memory engram in various ways. These manipulations include the ligand- and light-driven neuronal activation of neurons, pharmacological activators/suppressors of plasticity, combined with a variety of imaging techniques for the identification and exploration of the properties of cellular populations engaged in the long-term storage of memories (Guzowski et al., 1999; Zhang and Linden, 2003; Han et al., 2007; Reijmers et al., 2007; Silva et al., 2009; Bergstrom et al., 2011; Liu et al., 2012; Mayford, 2014; Lai et al., 2018).

The formation of memory engrams is associated with the action of multiple mechanisms that alter the functional properties of neuronal circuits during learning processes, which are collectively grouped as plasticity phenomena and act on multiple spatial and temporal scales (Bhalla, 2014). On the neuronal circuit level, plasticity is associated with changes in the intrinsic excitability of neurons (Zhou et al., 2009) and/or the excitability of their dendritic domains (Zhang and Linden, 2003; Losonczy et al., 2008), as well as on homeostatic phenomena that shape neuronal responses (Turrigiano, 2008). In addition, memory engrams are subject to the effects of epigenetic mechanisms such as the contribution of DNA methylation to memory storage (Day and Sweatt, 2011) and histone acetylation to memory maintenance and reconsolidation (Gräff et al., 2014). However, the main (and best studied) mechanism *via* which memories are believed to be encoded in these neuronal populations is the plasticity of synaptic strengths, which occurs primarily within the dendritic regions of excitatory neurons. At the level of the synapse, plasticity is expressed both presynaptically, and post-synaptically (Larkman et al., 1992), and even affects the local excitability properties of dendrites (Sjöström et al., 2008). Thus, to understand memory engram formation we need to understand how sub-cellular, primarily synaptic, modifications occur as a consequence of learning, and how these modifications relate to cellular engrams. It is important, first, to identify patterns of spatial allocation of synapses within dendrites during learning.

### Engram Allocation and Synapse Clustering

In the neocortex, most excitatory synapses reside on dendritic spines. Spines provide a conducive environment for synaptic plasticity and evidence shows that their emergence and stabilization correlates strongly with synaptic plasticity (Matsuzaki et al., 2004; Yasumatsu et al., 2008). Experiments have demonstrated spine dynamics such as emergence, retraction and morphological changes in multiple different brain regions (Trachtenberg et al., 2002; Holtmaat and Svoboda, 2009; Fu et al., 2012). The dynamics of spines, such as their turnover are affected by sensory experience and learning (Holtmaat et al., 2005; Hofer et al., 2009), and seem to be differentially regulated during adolescence and adulthood (Holtmaat et al., 2005; Zuo et al., 2005). Moreover, the location and distribution of stable spines is crucial for the activation of local ionic conductances, impacting the integration mode of dendrites (Ariav et al., 2003; Häusser and Mel, 2003; Poirazi et al., 2003a; Losonczy and Magee, 2006; Silver, 2010; Branco and Häusser, 2011; Yuste, 2011; Longordo et al., 2013) and consequently, the properties of cellular memory engrams. This distribution is greatly influenced by biophysical mechanisms within dendrites that shape postsynaptic responses and enable local forms of plasticity. The synapse strength modification that is required for plasticity can be causally linked to somatic spiking by the backpropagating action potential that reaches the dendritic branches (such as, in the case of Hebbian plasticity). In the absence of somatic spiking, that modification can also be induced locally *via* dendritic spikes (Hardie and Spruston, 2009).

Based on the above, there are at least two possible scenarios regarding the distribution of activated spines following experience and/or learning-induced plasticity: (a) random and distributed; or (b) patterned, in a way that sub-serves a specific function. We suggest that clustering is a type of patterned synaptic activation that is optimal for inducing local spiking and/or local synaptic plasticity. In the following paragraphs, we discuss experimental evidence for each of these scenarios.

#### Random and Distributed

Synapses can be scattered, seemingly randomly in dendrites, with the location of synapses being determined by anatomy (Braitenberg and Schüz, 2013). In this case, the connectivity of neuronal circuits depends on the overlap of dendritic arbors and axonal processes, as dictated by Peters’ rule (Peters et al., 1976). There is evidence that such random connectivity may exist in sensory cortices, however it is not known if this arrangement follows Peters’ rule. A series of experiments which used high-speed 2-photon imaging and electrophysiological recordings to probe the response properties of spines in the visual (Jia et al., 2010), auditory (Chen et al., 2011) and barrel (Varga et al., 2011) sensory cortical areas found that neighboring spines in dendrites of pyramidal neurons responded to different, apparently random orientations, sound frequencies or whiskers, and whisker combinations, respectively. This indicated that there was no specific regular organization of synapses at the spatial scale of the dendrite, but instead, there is an unstructured mixture of connections. Crucially, the authors did not observe dendritic regenerative events such as dendritic spikes in those studies. It can, therefore, be hypothesized that such observations support the idea that dendrites can act as simple conducting devices. This model of “passive” dendritic integration has inspired the earliest computational models of neuronal network research in the 20th century (Minsky and Papert, 1969; Hopfield, 1988). Even though such a view of dendrites is now outdated because it reduces neurons to point thresholding devices, they have been instrumental in the study of memory storage in artificial neuronal populations. These models have helped to established synaptic weight changes and connectionism as the predominant mechanism for learning in artificial systems (McClelland and Rumelhart, 1986; Hornik et al., 1989). Under such a model, the encoding of the engram would be expected to take place mainly *via* the dendritic backpropagation of action potentials, a plasticity process that does not require clustering.

#### Patterned

**Figure 1:**
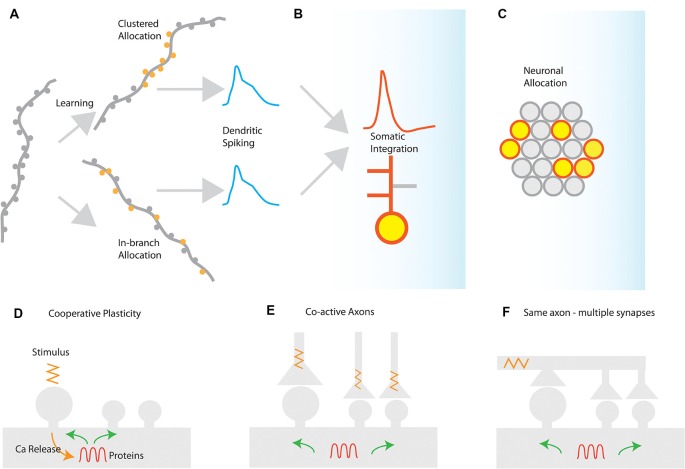
Synaptic and cellular engram formation. **(A)** Dendritic spikes can be induced either from localized activation of a co-active group of synapses (clustered synapse allocation) or from more dispersed activation of synapses within the same branch segment (In-branch allocation). **(B)** In both cases, the elicitation of dendritic spikes is integrated with the somatic compartment, controlling the action potential generation or bursting behavior of the neuron and enabling local or global plasticity to occur. **(C)** Neuronal populations activated for each memory are selected by mechanisms such as excitability and CREB activation leading to the selection of a population memory engram. *Mechanisms for the generation of synaptic clusters*: **(D)** cooperative sharing of plasticity-related resources such as proteins facilitates cooperative LTP in nearby synapses after LTP induction. The spreading of activation of plasticity-related proteins, enzymes, and mRNAs may prime nearby synapses for subsequent plasticity. **(E)** Co-active axons in nearby synapses can drive cooperative plasticity by initiating similar resource-sharing mechanisms as a result of coincident activation **(F)** a single axon may make multiple contacts in a short segment of a dendrite, thereby driving cooperative plasticity *via* synchronized synapse activation.

In several brain areas, it has been found that synapses are not randomly placed in dendrites, but tend to form groups or *clusters* of synapses (DeBello and Zito, 2017). These clusters of synapses have special significance for synaptic integration: activating a sufficient number of synapses in a short stretch of dendrite can elicit a self-regenerating, powerful dendritic spike (Losonczy and Magee, 2006). Dendritic spikes are much stronger and longer-lasting responses than ordinary excitatory post-synaptic potentials (EPSPs). They can strongly modulate the firing of the neuron, and can induce localized plasticity at the dendritic level (Spruston, 2008; Hardie and Spruston, 2009). It should be noted, however, that a dendritic spike will emerge under any condition that provides the critical (i.e., minimum-required), dendrite-specific, amount of local depolarization. A depolarization that is sufficient to open voltage-gated conductances and remove the Mg^++^ block from NMDA receptors *in its vicinity*, thus leading to supra-linear increases in Ca^++^/Na^+^ influx that rapidly surpass the threshold for Na^+^/NMDA/Ca^++^ spikes. There are at least two different ways to induce such critical dendritic depolarizations (Figure 1).

(a) Synapse clustering: nearly-synchronously activated inputs that are tightly-packed close to each other would be very efficient in both inducing a strong depolarization and counteracting signal attenuation at the site of EPSP induction (Figure 1A). In thick and long branches, signal attenuation is substantial and thus spatial clustering of inputs within small stretches is likely to be the most efficient way to induce a local spike. Such spatial clustering of inputs has been reported in several brain areas, either in its *anatomical* [inhomogeneous spine density (Makino and Malinow, 2011; Yadav et al., 2012; Druckmann et al., 2014)] or in its *functional* form [homogeneous spine density—clustered activation of inputs (Kleindienst et al., 2011; Fu et al., 2012; Takahashi et al., 2012)].
(b) In-branch localization of synapses: nearly-synchronously activated inputs that are distributed within short, thin branches are likely to have the same effect, as signal attenuation is much smaller in these branches (Figure 1A). Indeed, both experiments (Polsky et al., 2004; Losonczy and Magee, 2006) and modeling (Poirazi et al., 2003b; Gómez González et al., 2011) show that uniform distribution of co-activated inputs within the thin, oblique dendrites of CA1 pyramidal neurons is equally potent in generating dendritic spikes as restricting those activated inputs in small, spatially concise clusters. Such *in-branch* localization has been suggested to drive the tuning of visual cortex dendritic branches to specific orientations (Wilson et al., 2016) and is functionally analogous to the activation of a cluster of synapses.

Insofar as both synapse clustering and in-branch co-activation drive nonlinear responses in dendrites, their effect on the plasticity and function of the neuron is expected to be largely similar (Figures 1B,C). This may explain the disparity between experiments that have failed to find synaptic clusters of orientation in the visual cortex (Jia et al., 2010) with ones that do (Wilson et al., 2016).

## Mechanisms for the Generation of Synaptic Clusters

Considering the different patterns of spatial synaptic allocation that have been observed, it is important to identify mechanisms that lead to the nonrandom, clustered positioning of synapses. There are many mechanisms and/or conditions that can facilitate the formation of synaptic clusters in neuronal dendrites during learning and experience. In the following paragraphs, we discuss the most prominent ones, all of which require at least one of the following: (a) convergence of axonal projections carrying similar information onto the same dendrite; (b) the presence of active ionic conductances in dendrites; (c) activity-dependent axonal rewiring; and (d) local protein synthesis.

### Clustering During Development

During development, spontaneous activity refines neuronal connections, creating clusters of synapses with similar activity patterns. Niculescu et al. (2018) found that the activation of TrkB and postsynaptic brain-derived neurotrophic factor (BDNF) were required for synapse clustering in developing neurons. This intrinsic mechanism in developing hippocampal neurons acts in concert with proBDNF signaling, which downregulates synapses that are out-of-sync with the cluster activity. This combination suggests an efficient mechanism that facilitates the creation of synaptic clusters in the developing hippocampus. Lee et al. (2016) combined whole-cell electrophysiology with 2-photon imaging and glutamate uncaging to study the dynamics of synaptogenesis in developing hippocampal slices. They discovered that there NMDA receptor activation was functionally coupled with ryanodine-sensitive intracellular calcium release which is a crucial factor in the determination of the spatial and temporal dynamics of transient calcium signals which facilitate and lead to synaptogenesis. This mechanism is apparently tuned to detect synaptic inputs which are correlated in space and time, thus facilitating local synapse cluster formation. Thus, NMDA receptors, coupled to internal calcium store release, are well suited to guide synapse clustering and the fine structure of synaptic connectivity in emerging neural networks (Lee et al., 2016).

### Clustering by Cooperative Plasticity

Cooperativity is the characteristic property of LTP according to which the activated synapses can overcome the plasticity threshold as a group (Figure 1C). Cooperativity is believed to be initiated by the influx of calcium through NMDA receptor activation (Bliss and Collingridge, 1993; Sjöström et al., 2001). This activation and elevation of calcium levels initiates multiple biochemical signaling pathways in the dendritic domain (Baudry et al., 2011). Certain pathways can not only facilitate LTP at the stimulated synapse but also at neighboring synapses. This activity can coordinate the potentiation of multiple synapses leading to the formation of a group of nearby potentiated synapses, thus leading to the creation of a synapse cluster. The temporal dynamics of LTP and its consolidation (which can be slow as it is a protein-dependent process), allow for these interactions. Examples of these pathways are the MAPK (mitogen-activated protein kinase) and mTOR (mechanistic target of rapamycin) cascades, which are active for several minutes post-stimulation and LTP initiation (Wu et al., 2001). The long-time course of this activation allows proteins and kinases to spread to neighboring synapses, thus facilitating LTP induction in them. Part of the MAPK signaling pathway is the Ras GTPase, which has been correlated with increased spine volumes after LTP induction. Ras can spread to nearby spines over the time course of minutes (Harvey et al., 2008). Additionally, the RhoA GTPase similarly spreads out of stimulated spines for about 5 μm along the length of the dendrite (Murakoshi et al., 2011). The molecular mechanisms described are candidates to support the potentiation of groups of co-active synapses, which form synapse clusters at the spatial scale of about 20 μm (Hering and Sheng, 2001; Patterson and Yasuda, 2011; Winnubst et al., 2012). Glutamate uncaging experiments have directly demonstrated cooperative plasticity in dendrites *in vitro*, showing LTP cooperativity and the potential for clustering at the level of the single branch but not its siblings (Govindarajan et al., 2010).

Synaptic clustering is also dependent and supported by the presence of polyribosomes and smooth endoplasmic reticulum (SER). Using electron microscopy reconstruction, Chirillo et al. (2019) found that the rich presence of SER and polyribosomes in spines led to the creation of distinct synaptic clusters leaving the poor dendritic regions asynaptic. In addition, SER was enriched in the shafts near LTP-potentiated synapses, indicating that it might facilitate spine formation and potentiation in its vicinity in a cooperative manner.

### Clustering by Axonal Rewiring and Spine Turnover

In certain learning protocols, as well as during development, the addition of new synapses during synaptogenesis and spinogenesis tends to occur near existing, mature synapses (Fu et al., 2012). This addition of new synapses near existing ones effectively changes the wiring diagram and can lead to synapse clustering. Modeling studies (Poirazi and Mel, 2001) suggested a mechanism for activity-dependent rewiring according to which newly formed spines would establish stable contacts with passing axons, only if those axons had correlated activity with existing, mature spines, thus contributing to cluster formation. This mechanism relied on the formation of silent (NMDA-only) synapses that would turn into regular synapses in the presence of correlated input from nearby mature spines. Such changes are typically much slower than LTP cooperativity (on the order of days), as they require the restructuring of neural tissue and formation of new spines (Trachtenberg et al., 2002; Chklovskii et al., 2004; Holtmaat et al., 2005). Synapse turnover is a process that persists in the adult brain (Trachtenberg et al., 2002) and thus it is possible for LTP cooperativity to interact with synapse turnover or with the conversion of filopodia to mature dendritic spines. Such interaction would contribute to the *de novo* formation of synapse clusters.

It should be noted that clustering may also result from synapses originating from the same axon. Using serial-section transmission electron microscopy to image hippocampal tissue, Bloss et al. (2018) found single presynaptic axons that formed multiple spatially clustered inputs, These clustered synapses resided on the distal, but not on the proximal dendrites of CA1 neurons. These clustered synapses occurred predominantly in entorhinal rather than in thalamic afferents and had the morphological features of strong synapses. The authors verified using computational simulations that those clustered connections could efficiently depolarize the dendrites, thus sub-serving the same, common goal of clustering which is to engage the dendritic nonlinearities.

### Clustering (or in-Branch Localization) Reinforced by Dendritic Plateaus

Synaptic activation which leads to the generation of dendritic depolarization plateaus has been found to be effective for the initiation of synaptic potentiation, even in the absence of somatic spiking in CA1 neurons (Hardie and Spruston, 2009; Cichon and Gan, 2015). This mechanism could facilitate the formation or enrichment of synaptic clusters in specific dendrites, or more generally the in-branch potentiation of synaptic inputs within a dendritic segment, which in both cases increases the potential for somatic burst firing. Interestingly, dendritic voltage plateaus have been found to be correlated with the quick formation of new place fields in CA1 neurons (Sheffield and Dombeck, 2015; Bittner et al., 2015) and were produced *via* interaction between entorhinal and hippocampal CA3 input. Interestingly, these plateaus are implicated in a non-Hebbian plasticity rule that allows the formation of place fields in CA1 neurons primed by dendritic plateaus which occurred many seconds earlier, a novel form of plasticity termed *behavioral time scale synaptic plasticity* (BTSP; Bittner et al., 2017). It is thus possible that clustering, which can naturally cause the initiation of dendritic plateaus is correlated with this form of plasticity.

In parallel with excitatory synapse clustering, recent research has identified that inhibitory synapse allocation may also follow similar dynamics. When dendritic spines undergo modification *in vivo*, it was found that inhibitory synapses located within 10 μm of them also undergo modifications, and this spatially coordinated clustering of excitatory and inhibitory synapses modifications was increased under sensory deprivation. (Chen et al., 2012). This could indicate a “co-clustering” mechanism that allows inhibitory synapses to regulate the plasticity or the function of excitatory synaptic clusters. Indeed, the loss of inhibition in a region may facilitate excitatory synapse clustering. Computational modeling has suggested that inhibitory synapses can determine the sign and presence of plasticity in neighboring dendritic segments (Bar-Ilan et al., 2013). Evidence from *in vitro* experiments indicates that GABA uncaging reduces the calcium transients caused by backpropagating action potentials within a distance of 20 μm from the uncaging site, indicating that appropriate placement of inhibitory dendritic synapses may have a crucial role in permitting the formation of synaptic clusters nearby (Hayama et al., 2013).

## Synapse Clustering Enables New Modes of Neural Processing

The machinery of clustering and its observation in many different experiments raise intriguing questions about the functional role of clustering in the brain. The now abundant evidence of dendritic spike generation, patterned spatial synaptic arrangement, and clustered activation both *in vitro* (Häusser et al., 2000; Schiller et al., 2000; Ariav et al., 2003; Losonczy and Magee, 2006; Nevian et al., 2007; Larkum and Nevian, 2008; Kim et al., 2012; Makara and Magee, 2013) and *in vivo* (Lavzin et al., 2012; Major et al., 2013; Smith et al., 2013) highlight that dendrites have computational capabilities that go beyond the simple linear integration which is mediated by distributed connectivity. This means that the connectionist models which account only for distributed connectivity needed to be updated to account for the effect of nonlinear dendrites.

**Figure 2:**
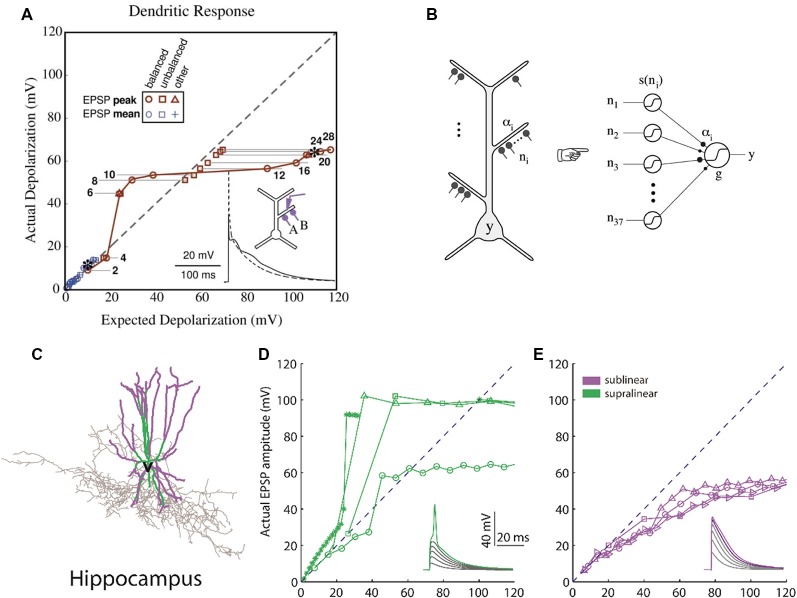
Computational basis of 2-layer neuronal integration. **(A)** Nonlinear integration in CA1 neurons can results in a sigmoidal-like response of the dendrite to synaptic input. y-axis: observed depolarization (excitatory post-synaptic potentials, EPSP), x-axis: expected depolarization (EPSP) if the dendrite had a completely linear response. **(B)** The simplified model which predicts the firing rate output of a CA1 neuron is a 2-layer neural network with sigmoidal activation functions [adapted from Poirazi et al. (2003a,b), with permission]. **(C)** Hippocampal fast-spiking interneurons (the morphology shown) also have nonlinear dendrites. **(D)** Dendrites with supralinear responses axes as in **(A)**. **(E)** Dendrites with sublinear responses. Adapted with permission from Tzilivaki et al. (2019).

Computational studies of clustered activation of synaptic inputs in the dendrites of models of both simplified (Mel, 1993; Poirazi and Mel, 2001) and also anatomically and biophysically detailed neurons (Poirazi et al., 2003a,b) have demonstrated how clustered input can engage local dendritic nonlinearities to influence, not only the electrical effect of the dendrite, but also the neuronal output. The synchronous activation of synapses which reside in the same apical branch of a pyramidal neuron model resulted in supralinear responses (Poirazi et al., 2003a). This computational prediction was verified *via* recordings from L5 neocortical pyramidal neurons (Polsky et al., 2004). The supralinear response of the neurons is the result of the dendritic spikes initiation, a phenomenon that is not present when synapses are stimulated between different branches. Supralinear dendritic responses have been reported in experiments in oblique dendrites of CA1 pyramidal cells by stimulating groups of synapses within individual radial oblique dendritic branches (Losonczy and Magee, 2006). This ability to respond to synchronized input indicates that supralinear dendrites may act as coincidence detectors *via* the initiation of fast dendritic spikes (Ariav et al., 2003; Losonczy and Magee, 2006; Gómez González et al., 2011) or may detect asynchronous bursting inputs *via* the initiation of slow dendritic spikes (Gómez González et al., 2011). The nonlinear activation of such independent integrative compartments has established the two-stage model of neuronal processing (Figure 2), according to which dendrites act as a second layer of nonlinearities, and thus each neuron can be considered as a two-layer neural network (Poirazi et al., 2003b; Katz et al., 2009). This finding has profound implications for the functioning of neurons, primarily because it increases their storage capacity by an order of magnitude (Poirazi et al., 2003a). Interestingly, recent work has found that interneurons can also exploit dendritic nonlinearities of their dendrites (Figure 2), which allows them to be modeled as a 2-layer network, thereby increasing their computational capacity (Tzilivaki et al., 2019). It is interesting thus to explore how the brain takes advantage of the capacities afforded by clustering and its dendritic machinery, and this has been a focus of experimental dendritic research in recent years.

## Synapse Clustering in Sensory Systems

Synapse clustering is relatively well studied in sensory cortices, and multiple functions have been ascribed to the nonlinear properties of clustered synapses. In fact, the first evidence for anatomical synapse clustering in relation to experience was demonstrated in the dendrites of the cells of the adaptive microcircuit of the auditory localization circuit of the barn owl (McBride et al., 2008). Barn owls which have been raised with prismatic spectacles typically develop an adaptive zone. This does not exist in normal animals and is a result of the animal’s abnormal experience. McBride et al. (2008) found increased clustering of axodendritic contacts in the adaptive zone as well as decreased clustering in the normal zone, indicating that this type of learning is correlated with dendritic synapse clustering. In another study, Takahashi et al. (2012) visualized the synaptic activation of neurons in the barrel cortex of mice and found that activated synapses formed functional “assemblets.” These synapses were synchronized and spatially confined (Takahashi et al., 2012). Neighboring spines were found to be significantly co-activated compared to control and formed functional synaptic assemblets which consisted of groups of synapses (2–12 synapses spaced <10 μm) in dendritic branches. The spines of the assemblets were larger in size in comparison to spines that did not participate in assemblets, indicating that assemblets were likely formed *via* LTP mechanisms. The authors confirmed that by demonstrating the reduced clustered activity of synapses when the tissue was cultivated in the presence of NMDA receptor antagonist. The authors were able to pinpoint the source of the observed clustered co-activation of synapses, which was attributed to the concurrent activation of the afferent axons terminating on the clustered synapses.

In the visual cortex, Wilson et al. (2016) used 2-photon *in vivo* calcium imaging to characterize the orientation tuning and arrangement of synaptic inputs terminating on spines of Layer 2/3 pyramidal neurons in the ferret visual cortex. Interestingly, while the orientation preference of the visual cortex neuron represented the summed synaptic orientation-tuned inputs to the neuron, it did not account for differences in orientation selectivity among neurons. The authors identified a nonlinear relationship between input and outputs that was correlated with the spatial clustering of co-tuned inputs. Dendritic branches with clusters of similarly tuned inputs exhibited calcium dendritic events, which in turn shaped the orientation selectivity of the neuron. Thus the degree of clustering on single visual neurons strongly predicts somatic orientation selectivity. Along the same lines, Gökçe et al. (2016) mapped the spatial organization of glutamatergic synapses *between* layer 5 pyramidal in the mouse visual cortex using a combination of optogenetics and 2-photon calcium imaging. The authors performed a combinatorial analysis of the likelihoods of specific synapse arrangements, to demonstrate that the synapses of intralaminar inputs formed clusters on the *basal* dendrites of layer 5 pyramidal cells, containing 4–14 synapses in a stretch of ≤30 μm of the dendrite (Gökçe et al., 2016).

In order to identify the role of clustered inputs in the visual system, Iacaruso et al. (2017) used 2-photon imaging to map the spatial receptive fields in dendritic spines the visual cortex of mice. This allowed them to determine which synaptic inputs responded to different locations in the visual scene. Their results showed that inputs that represented similar visual features from the same location in visual space clustered on neighboring spines, while inputs from visual field regions outside the receptive field of the neuron tended to synapse on higher-order branches. Those long-range inputs were more also likely to represent the same oriented edges as the postsynaptic neuron when the receptive field of the input was spatially displaced in the direction of the axis of the receptive field orientation of the postsynaptic neuron. This specific synaptic connectivity is apparently suited to amplify the responses to elongated edges. These edges are enriched in the natural visual environment, and thus this arrangement of clustered and non-clustered synapses could be a mechanism for contour integration.

In addition to experimental work, the potential of synapse clustering to determine the neuronal output of sensory neurons has been investigated using computational models. Nonlinear dendritic processing can affect the stimulus selectivity of a neuron, even if the total synaptic weight of the preferred stimulus does not exceed that of the non-preferred input. Cazé et al. (2017) used computational modeling to demonstrate how this stimulus selectivity arises from the spatial distribution of synapses. This stimulus selectivity increases the neuron’s robustness to synaptic and dendritic failure. Using compartmental modeling they also showed how this clustered activation model is compatible with the that the mixture of selectivities in dendrites is different from the somatic selectivity (Cazé et al., 2017). Synaptic clustering may also have a functional role in the olfactory system. Migliore et al. (2015) used a 3D model of mitral and granule cell interactions, to identify the mechanisms for the formation of multiple odor-activated synaptic clusters related to individual glomeruli. They studied how and to what extent the glomerular units interact or interfere with each other during learning, and developed a theoretical framework in which the olfactory bulb contains “odor operators” unique to each individual. While speculative, this model provides a function for clustering in the olfactory system (Migliore et al., 2015).

Overall, clustering in sensory systems is relatively well studied and we now have a partial understanding of how it contributes to sensory tuning. Considering the long timescale of sensory system development, it is likely that developmental mechanisms of clustering are primarily responsible for sensory synaptic clusters. Indeed, one of the first observations of clustering was in the barn owl visual adaptive region during development (McBride et al., 2008).

## Synaptic Clustering in Learning and Memory

Studies of learning and memory have recently ascribed multiple memory-related roles to synapse clustering. Makino and Malinow (2011) used fluorescent tagging of glutamate receptor type 1 (GluR1) subunits to visualize the trafficking of AMPA receptors during the normal sensory experience and also during periods of sensory deprivation (whisker removal) in mice. Normal experience (e.g., whisking) caused coordinated trafficking of GluR1 subunits to neighbor synapses in the dendrites of somatosensory neurons. The clustering of GluR1 subunits was abolished on sensory-deprived mice, which indicates that learning under a rich sensory environment resulted in higher clustering. Kramár et al. (2012) examined the role of timing in the induction of LTP in rat hippocampal slices in order to explore the possible effects of spaced repetition in learning. Interestingly they found that when theta stimulation was spaced apart by more than 1 h there was a marked enhancement of LTP. The effect of spaced repetition resulted in the potentiation of co-localized synapses which did not undergo LTP initially. This suggests a general rule for clustered potentiation as a mechanism that results from spaced repetition and improves memory learning (Kramár et al., 2012).

**Figure 3:**
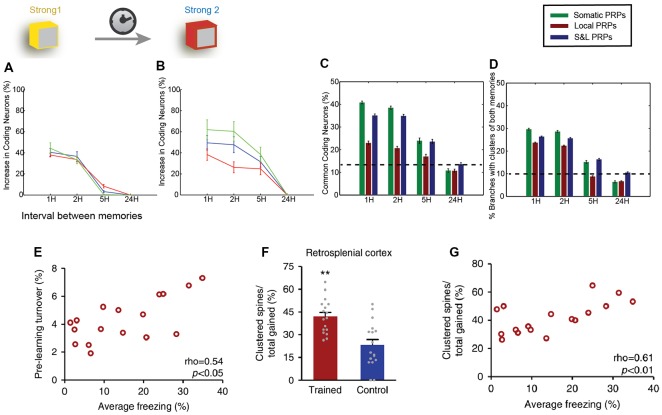
Clustering in memory. Computational modeling suggests that two memories that overlap temporally and thus have the ability to share proteins and resources *via* cooperative plasticity and synaptic tagging, are co-allocated in overlapping neuronal populations. **(A)** Increase in the size of memory engram (neuronal populations active during recall) of the first memory when paired with a second one as a function of the interval between the two memories (x-axis). LTP cooperativity enables enhanced allocation of the memory for the first 2 h. Panel **(B)** as in **(A)**, but for the second encoded memory. The increased excitability of the neurons encoding the first memory enables enhanced allocation of the second memory for up to 5 h. **(C)** The two memories being encoded are allocated in overlapping populations of neurons, and the overlap varies both as a function of the interval between memories (x-axis) and dependent on whether protein synthesis is limited to a single branch, or the entire neuron, or both (color coding). **(D)** In addition to overlapping populations, memories are also encoded in overlapping dendrites, i.e., they create clusters together, dependent on time and locus of protein synthesis similarly to **(C)**. **(E)** Imaging of retrosplenial cortex dendrites shows that pre-training synaptic turnover in a stretch of dendrite correlates with the performance of contextual fear memory after a learning task. **(F)** The learning task resulted in a higher incidence of clustered synapses post-training compared to controls. **p < 0.01. **(G)** Increased clustering of synapses gained post-training was found to correlate with behavioral performance. Panels **(A–D)** adapted from Kastellakis et al. (2016), with permission. Panels **(E–G)** adapted from Frank et al. (2018), with permission.

Localized synaptic turnover has also been found to have an effect on both memory performance and synapse clustering. Frank et al. (2018) used *in vivo* 2-photon microscopy to trace the spine dynamics in dendrites of the retrosplenial cortex before and after episodic-like memory learning. They found that increased turnover rates before learning correlated with post-learning performance of the learned task (Figure 3E) as well as with increased learning rate. Interestingly, pre-learning turnover and memory performance also correlated with an increased incidence of synapse clustering in the imaged dendrites (Figures 3F,G), even though the total number of newly acquired synapses did not change. In addition, the authors used genetically modified CCR5-knockout mice, which are known to exhibit enhanced spine turnover, and demonstrated that it results in enhanced memory and spine clustering of new synapses. Detailed analysis showed that there are sub-regions of increased synaptic turnover (hotspots) within dendrites, which facilitate the incidence of synaptic clusters post-learning. It thus becomes evident that these hotspots of increased synaptic turnover in dendrites serve as a substrate for memory-enhancing synapse clustering.

Last but not least, computational modeling has been instrumental for the study of the relationship between synapse clusters and memory. In fact, models were the first to identify the potential for nonlinear integration and processing in dendrites (Segev and Rall, 1988; Mel, 1993; Poirazi and Mel, 2001), informing subsequent electrophysiological and imaging studies. Recent results suggest that the engram formation process is guided by a competitive selection process in which neurons are recruited to encode a memory according to their CREB activation and intrinsic excitability levels (Han et al., 2007). Using computational modeling, it was shown that this CREB-dependent excitability in cooperation with the process of synaptic tagging and capture leads to clustered potentiation of synapses in dendrites (Kastellakis et al., 2016). When multiple memories are stored in the same circuit, co-clustering of related memories occurs, along with neuronal co-allocation (Figures 3C,D), and thus synapse clustering might provide a substrate for linking memories into memory episodes (Figures 3A–D). In addition, branch-specific learning rules which depend on the elicitation of dendritic spikes (and thus on the clustering of synapses) can induce competition between different dendritic branches, allowing neurons to bind different memory features in different branches (Legenstein and Maass, 2011), thus strongly enhancing the processing capabilities of individual neurons (Poirazi and Mel, 2001).

Overall, clustering interacts with learning and memory in ways that extend beyond Hebbian learning and enable new modes of processing. Cooperative plasticity leading to clustering is likely to have a major contribution to learning and storage of information. It has been thus suggested that the dendritic branch, due to its unique electrical integration properties and its ability to support clustered synapses, is the fundamental unit of processing and memory allocation in the brain (Govindarajan et al., 2006; Branco and Häusser, 2010).

## Synapse Clustering and Pathologies

Could synapse clustering play a role in pathological states and disease? Studies of synapse clustering throughout the years have attempted to identify the ways in which the brain exploits the functional properties of synaptic clusters. Theoretical considerations have suggested that synapse clustering, as a mechanism of associativity, might be disrupted in pathological cases such as autism and schizophrenia. It was speculated for example that over-clustering of synapses representing different memories could lead to confusion of memories and remote associations as seen in schizophrenia, while under-clustering might reduce the relatedness between memories and knowledge domains, evident in autism (Kastellakis et al., 2015).

Recent studies have shed light on the relationship between abnormal dendritic clustering of synapses and their putative relationship to pathological states. Ash et al. (2018) found that repetitive motor learning in a mouse model of MECP2-duplication syndrome (which is a high-penetrance form of syndromic autism) increased the stabilization of dendritic spines following learning. This result is the reverse of what was earlier speculated, but nevertheless highlights a connection between clustering and autism. The ERK signaling pathway was found to be hyperactive after learning, leading to an increase in the number of clustered synapses being formed, and predicted enhanced motor learning. This increased clustering could thus be implicated in behaviors that are reminiscent of savant-like behaviors which are associated with autism (Ash et al., 2018). In a study of the effect of an early-life intervention on adulthood behavior, Skilbeck et al. (2018) assessed the relationship between early-life anxiety and the clustering of GABAa receptors. They found that animals which showed increased anxiety-type behavior in an elevated-plus maze experiment also showed reduced colocalization of GABA_A_ a2 subunits relative to control animals, suggesting that early-life environment may lead to long-term changes in adulthood behavior *via* its effect in the clustering of GABAa receptors. Thus reduced GABAA receptor synapse clustering may underlie enhanced anxiety (Skilbeck et al., 2018). Finally, in a mouse model of Huntington’s disease, Murmu et al. (2013) found that the density and the stability of dendritic spines were reduced compared to controls, even though the spine formation rate was overall increased. This may indicate that the dynamics of dendritic spines, which directly affect synapse clustering, may have a role in the early symptoms of Huntington’s (Murmu et al., 2013).

Overall, these reports suggest a role for synapse cluster disruptions in mental disease. It’s likely that developmental mechanisms of plasticity may be of particular relevance to these lifelong diseases, although the genetic changes associated with pathologies may indicate that any mechanisms that lead to clustering may be affected.

## Perspective

There is now abundant evidence that synaptic arrangements in dendritic branches are not randomly created. Mechanisms that favor clusters of synapses are active already in the developmental stage, and multiple plasticities, learning and memory processes also facilitate the formation of synaptic clusters. It is thus evident that the brain does take advantage of the computational power of dendrites, *via* synapse clustering, and exploits it to perform various brain functions. Given the disparity of findings regarding the effects of learning on synapse distribution and activation properties, it is imperative to have the appropriate tools to establish causal links between learning and spine/synapse changes in engram studies.

Early modern studies of the memory engram used reporters for the expression of immediate early genes such as *cFos* or *Arc* to identify the neurons that constitute the memory engram (Guzowski et al., 1999). These tools did not allow a detailed mapping of the synaptic and dendritic features of engram neurons. Newer tools employ more precise, temporally detailed mapping of calcium transients which are related to synaptic activity. The recently developed photoconvertible activity reporter CaMPARI (calcium modulated photoactivatable ratiometric indicator) allows the temporally precise measurement of calcium levels generating a snapshot of evoked activity such as zebrafish larvae (Fosque et al., 2015) and has been extended to the measurement of sub-threshold synaptic activity. Another photoconvertible marker called SynTagMA is being developed which can precisely mark synapses within a ~2 s time window, allowing the distinction of both pre-synaptically active axon terminals and post-synaptically active synapses (Perez-Alvarez et al., 2019). In addition, glutamate sensitive reporters such as iGluSnFR can be used to monitor the activity of presynaptic terminals *via* fluorescence microscopy (Marvin et al., 2013). Such methods will allow scientists to extend the study of the structure of neural engrams beyond the neuronal and population levels, allowing the mapping of the fine structure of memory traces in dendritically-located synapses.

The exploration of the ways in which clustering may contribute to computation in the brain is also an open field of theoretical research. The findings in the field so far indicate that dendrites are key structures in a wide range of phenomena, and their nonlinear capabilities are optimally engaged by synapse clustering. For example, dendritic nonlinearities increase the storage capacity of neurons (Poirazi and Mel, 2001), specialize dendritic feature binding (Legenstein and Maass, 2011), determine angular and spatial properties of visual cortex neurons (Lavzin et al., 2012; Iacaruso et al., 2017), strengthen learning and memory (Frank et al., 2018), contribute to binding of memories in episodes (Kastellakis et al., 2016), and generally contribute to sensory tuning (Migliore et al., 2015; Cazé et al., 2017). The finding that dendritic nonlinearities enable both excitatory and inhibitory neurons to act as 2-layer integrators (Poirazi et al., 2003b; Tzilivaki et al., 2019) may enable neurons to perform computations that are hitherto unknown. The clustering of synapses facilitates this engagement of nonlinearities by binding together information effectively without requiring other resources. This field of clustered and two-stage integration, in fact, presents a vast field for theoretical work to explore, and it will be interesting to discover new ways in which neurons exploit these synaptic arrangements to improve function. Alongside theoretical studies, experimental results keep unraveling these phenomena, adding to what is already a vast body of experimental literature on detailed processing at the synaptic and dendritic levels. These findings are not yet incorporated into mainstream connectionist models of learning, and are only beginning to be incorporated in machine learning and deep learning models (Guergiuev et al., 2016), where we envision new and important roles in solving challenging tasks.

## Author Contributions

GK and PP conceived and wrote the manuscript.

## Conflict of Interest

The authors declare that the research was conducted in the absence of any commercial or financial relationships that could be construed as a potential conflict of interest.
